# Glomerular filtration rate determination by creatinine and cystatin-C in patients with acute pyelonephritis

**DOI:** 10.22088/cjim.9.3.290

**Published:** 2018

**Authors:** Hadi Sorkhi, Raheleh Behzadi, Neda Joghtaei, Mohammad Poornasrollah, Ali Bijani

**Affiliations:** 1 Non-Communicable Pediatric Diseases Research Center, Health Research Institute, Babol University of Medical Sciences, Babol, Iran; 2 Student Research Committee, Babol University of Medical Sciences, Babol, Iran; 3 Social Determinants of Health Research Center, Health Research Institute, Babol University of Medical Sciences, Babol, Iran

**Keywords:** Nephrotoxicity, Amikacin, Cystatin C, Creatinine, Acute pyelonephritis, Glomerular filtration rate

## Abstract

**Background:**

Measurement of glomerular filtration rate (GFR) and monitoring of it in any patient on nephrotoxic drugs is very important. Recently, cystatin C (cys-C) has been introduced as a better marker for determining and monitoring renal function than creatinine especially in a mild decrease of GFR. This study was done to assess the change of GFR measurement based on serum Cys-C and creatinine and their comparison in children with acute pyelonephritis on amikacin.

**Methods:**

All children with acute pyelonephritis who were admitted in Nephrology ward were enrolled in this study. Serum creatinine, serum cys-C and the GFR calculation based on them were measured in patients on the day of admission (day zero) and then on days 3 and 7 after the start of treatment with amikacin and p-value less than 0.05 was considered significant.

**Results:**

Among the 70 children, 61 patients were females and the others were males. Mean age was 42.66±41.53 months. Estimated GFR based on creatinine on day 0 (before amikacin administration), 3 and 7 were 72.41±20.89 ml/min/1.73 m^2^, 78.42±21.15 ml/min/1.73 m^2^ and 80.5±22.43 ml/min/1.73 m^2^, respectively. Moreover, GFR based on cys-C during these days were 116.23±58.9 ml/min/1.73 m^2^, 116.49±53.31 ml/min/1.73 m^2^ and 108.37±51.02 ml/min/1.73 m^2^, respectively (p<0.05).

**Conclusions:**

According to this study, decrease of GFR calculation based on Cys-C was seen and estimated GFR was not changed according to creatinine. So, we recommend the use of cys-C for the monitoring of renal function in any patient treated with nephrotoxic drugs such as amikacin.

Renal function is determined by glomerular filtration rate (GFR). Measurement of GFR by inulin is a gold standard method. Of course clearance of iohexol is a good method for GFR calculation (1). The calculation of GFR by these methods was limited by need of high specialized equipment and personnel and also was very expensive. So, the measurement of GFR by these materials is used only for researchers (2, 3). In practice, the most common method for calculation of GFR is creatinine clearance. Because creatinine is an endogenous substance and its measurement is available and low-cost. Creatinine is produced via metabolism of the muscles. The main excretion route of creatinine is glomerular filtration. Besides, the serum level of creatinine could be affected by tubular secretion, sex, age, hepatic disorder, malnutrition and muscle mass (4-6). Recently an endogenous marker for estimation of GFR is cystatin C (cys-C). It is a low molecular weight protein and reabsorbed and catabolized by the proximal tubules. It is removed from the circulation by glomerular filtration.

GFR estimation by Cys-C is not influenced by age, gender, inflammation, muscle mass and other variables that can have effect on serum creatinine concentration. Cys-C seems to be a more accurate marker for drug dosage adjustment during medication, especially in a mild decrease of GFR in children (2, 7-12).

Aminoglycoside antibiotics are used for treatment of many infections, especially gram-negative bacteria and can be used in children with pyelonephritis (13-15). The main route of excretion of aminoglycosides is the kidneys. Aminoglycosides are transferred from plasma into the urine by glomerular filtration. Thus, accurate kidney function monitoring during the consumption of these drugs is necessary. There is a risk of renal nephrotoxicity during the use of aminoglycoside. The risk of toxicity is influenced by underlying renal disease, dose of drug, duration of use, and the patient's age (16-18).

This study was done in pediatric patients with pyelonephritis that were treated with amikacin and its effect on renal function was compared by GFR estimation between the clearance of creatinine and Cys-C.

## Methods

A total of 70 children (61 girls and 9 boys) with acute pyelonephritis were enrolled in the study and their age was between 2 months to 14 years old (42.66±41.53 months). Pyelonephritis was diagnosed with fever, positive urine culture and pyuria in the urine analysis. All patients were admitted in the Nephrology ward of the Amirkola Childrens Hospital, Babol (north of Iran). According to our previous study, the most sensitive parenteral drug for treatment of UTI was amikacin (19).

 So in this study, all patients with diagnosis of pyelonephritis were treated with amikacin(19). All patients were treated with a dose of 5mg/kg Amikacin intravenously every 8 hours for 7 days. Initial renal function was normal in all patients. 

All patients with positive history of increased creatinine or kidney disease were excluded from the study. For every patient, serum creatinine and Cys-C were measured before the amikacin administration (day 0) and on days 3 and 7 after the start of treatment. GFR was calculated based on these indicators during the treatment period. Creatinine clearance was calculated according to the Schwartz equation (4). Cys-C clearance was calculated according to Filler formula (20). Patients were divided into two groups according to their age (patients younger than 2 years and those older than 2 years of age). Statistical analysis was performed using the SPSS 17 statistical software. Also, paired t-test, repeated measures ANOVA and the Pearson correlation coefficient were used. A p<0.05 was considered statistically significant.

## Results

Among the 70 patients, 61 (87.1%) were females and (12.9%) were males. Mean age was 42.66±41.53 months. Thirty eight (54.28%) patients were less than 2 years old and 32 (45.71%) patients were more than 2 years. The height of patients ranged from 50 cm to 143 cm (88.56±25.82 cm). Serum level of blood urea nitrogen (BUN), creatinine and Cys-C were measured on the admission day or zero (before amikacin administration), days 3 and day 7 after the initiation of treatment. Patient's BUN on days 0, 3 and 7 were 9.22±2.43 mg/dl, 8.97±2.53 mg/dl and 9.31±2.76 mg/dl, respectively. According to table 1, serum creatinine and Cys-C levels were 0.46±0.09 mg/dl and 0.90±0.27 mg/l on day 7, respectively.

Creatinine clearance (GFR) during the amikacin treatment, according to the Schwartz formula was 96.37±27.04 ml/min/1.73m² on day 7. Also, estimated GFR based on Cys-C on day 7 was 108.37±51.02 ml/min/1.73m² (p<0.05) (table 1).

According to the age, the patients were divided into two groups: patients less than 2 years and those older than 2 years of age. Hence, in the group aged under 2 years, creatinine concentration in serum on days 0, 3 and 7 was 0.44±0.07 mg/dl, 0.41±0.06 mg/dl and 0.41±0.5 mg/dl and Cys-C was 0.98±0.28 mg/dl, 0.96±0.26 mg/dl and 1.01±0.23 mg/dl on these days, respectively. In this group GFR estimation based on creatinine on day 7 was 80.34±23.75 ml/min/1.73m² and GFR calculation based on Cys-C was 88.84±36.30 ml/min/1.73m². (p>0.05) (table 2). 

In children more than 2 years old, creatinine concentration in serum on days 0, 3 and 7 was 0.58±0.10 mg/dl, 0.54±0.08 mg/dl and 0.52±0.08 mg/dl and Cys-C was 0.74±0.27 mg/dl, 0.72±0.24 mg/dl and 0.77±0.27 mg/dl, respectively. GFR estimation based on creatinine on day 7 was 115.40±16.31 ml/min/1.73m² and GFR calculation based on Cys-C was 131.57±56.56 ml/min/1.73m² on day 7 (p<0.05) (table 2).

**Table 1:** Mean, standard deviation and range of serum Bun level, creatinine, Cystatin C, GFR based on creatinine and GFR based on Cystatin C in children with pyelonephritis treated with Amikacin

**Day and variation**		**Serum Creatinine** **(mg/dl)**	**GFR** * ** based on** **Creatinine** **(ml/min/1.73 m²)**	**Serum** **Cystatin C** **(mg/l)**				**GFR based on** **cystatin C** **(ml/min/1.73 m²)**			
Mean ± SD	0	0.62 ± 0.13	72.41 ± 20.89	0.87 ±0.30			116.23 ± 58.90			
Range		0.31-1.09	38.63-141.46	0.352-1.718		36.70-360.40			
Mean ± SD	3	0.58 ± 0.11	78.42 ± 21.15	0.85 ± 0.28			116.49 ± 53.31			
Range		0.31-0.95	37.35-139.14	0.345-1.607	40.40-272.80			
Mean ± SD	7	0.57 ± 0.11	80.50 ± 22.43	0.90 ± 0.27			108.37 ± 51.02			
Range		0.36-0.82	37.35-144.47	0.390-1.634	39.40-310.90			

* Glomerular filtration rate

**Table 2:** Mean, standard deviation and range of serum creatinine, Cystatin C, GFR based on creatinine and GFR based on Cystatin C in children younger and older than 2 years with pyelonephritis treated with Amikacin

	**Day and variation**		**Serum Creatinine** **(mg/dl)**	**GFR based on** **Creatinine** **(ml/min/1.73 m²)**	**Serum Cystatin C** **(mg/l)**	**GFR based on** **cystatin C** **(ml/min/1.73 m²)**
Younger than 2 years	0	Mean ± SD	0.44±0.07	74.14 ± 23.04	0.98 ± 0.28	93.26 ± 39.16
		Range	0.25-0.63	38.63-132.62	0.42-1.71	36.70-200.30
	3	Mean ± SD	0.41± 0.06	78.99 ± 23.10	0.96 ± 0.26	97.26 ± 45.72
		Range	0.25-0.56	37.35-132.62	0.42-1.60	40.40-218.80
	7	Mean ± SD	0.41± 0.05	80.34 ± 23.75	1.01 ± 0.23	88.84 ± 36.30
		Range	0.29-0.57	37.35-120.37	0.54-1.52	43.60-193.90
Older than 2 years	0	Mean ± SD	0.58 ± 0.10	104.21± 15.56	143.50 ± 66.92	0.74 ± 0.27
		Range	0.36-0.88	78.68-141.21	54.10-216.40	0.35-1.31
	3	Mean ± SD	0.54 ± 0.08	111.66 ± 15.69	139.32 ± 53.28	0.72 ± 0.24
		Range	0.33-0.77	72.35-139.14	68.00-211.20	0.34-1.11
	7	Mean ± SD	0.52 ± 0.08	115.40 ± 16.31	131.57 ± 56.56	0.77 ± 0.27
		Range	0.36-0.66	78.68-144.47	39.40-218.90	0.38-1.63

## Discussion

According to this study, during the 1-week treatment with amikacin in children with acute pyelonephritis, serum creatinine and calculating GFR did not significantly changed. But, the GFR that was calculated by Cys-C changed and decreased during the 1- week treatment with amikacin (p<0.05). 

In clinical practice, GFR based on creatinine calculated with Schwartz formula is used. Furthermore, this GFR calculation may be overestimated in comparison to the gold standard method (clearance of inulin). As a consequence, we must use a better marker that is endogenous, non-expensive and non-affected by many factors such as age, drugs and so on. These factors affect GFR calculation with creatinine. Cys-C is a protease inhibitor that is responsible for the intracellular catabolism produced by all nucleated cells. It is completely filtered from the glomerulus and reabsorbed and metabolized by the tubules. Cys-C is independent of inflammatory process in the body, muscle mass, age, sex and nutritional status and does not cross the placenta. It is used calculating GFR in adults since 1985 by Simonsen (12, 21-24). There are some studies to compare GFR calculation based on creatinine and Cys-C. Although there were no studies on children with UTI (urinary tract infection) and on amikacin Tsujita study was done on 73 patients with kidney transplant and mild to moderate impaired GFR and compared calculated GFR based on Cys-C and creatinine with inulin. This study showed that Cys-C can determine GFR more accurately than creatinine clearance in these patients (25). Besides, calculating GFR based on Cys-C in adult patient with chronic kidney disease (CKD) had better accuracy than creatinine to determine GFR (26). The same result was shown by Linen in children with impaired renal function (7). 

As mentioned above, GFR based on Cys-C has better accuracy than creatinine and may replace the calculation of GFR instead of creatinine. But there are limited studies to evaluate drug nephrotoxicity and compare the change and probability decrease of GFR by Cys-C. Conseqently, further studies need to be done. Halacova reported 71 cystic fibrosis patients who were treated with amikacin. GFR in patients was determined for several days. Finally, he showed Cys-C is more appropriate to determine the change of GFR than creatinine (17). In another study of 130 patients treated with amikacin, tobramycin, vancomycin and gentamicin, reported that Cys-C is more valuable than creatinine for calculation of GFR and drug dose adjustment (27). In our study, a GFR calculation based on creatinine did not change during the use of amikacin in children with pyelonephritis after 7 days. But, according to the calculation of GFR by Cys-C in these patients, GFR statistically decreased. 

Although this reduction of GFR may not be clinically important But, a more serious decrease of GFR may occur with increased duration of treatment or higher dose of drug. This study showed there is a risk of change of GFR even in a short period use of the drug, especially in children treated for acute pyelonephritis. There are some studies that showed different results. O'Riordan’s study concluded that serum Cys-C does not have any advantages over creatinine in elderly people in predicting digoxin clearance. But only 18 volunteers completed the study. Definitely, the sample size was small (28). Schuck et al. also showed no considerable difference between serum Cr, CysC and GFR based on Cockroft-Gault formula for evaluating GFR and adjusting dosage of drug in adults (29). 

Naturally, there are some aspects that may be considered for the estimation of GFR based on Cys-C. For example, there is not any study about the effect of tubular dysfunction at serum level of Cys-C and also on GFR. The serum level of Cys-C may be affected by CRP, thyroid dysfunction and corticosteroid consumption. Moreover, compared to GFR with creatinine and Cys-C in children may be problematic and in compatible. There are some reports about the effect of age, sex and weight on serum level of Cys-C (30-35). According to the risk of amikacin nephrotoxicity in children with low ages, the patients were divided into two groups: under and above the age of 2 years, respectively. In both groups, serum Cr and GFR based on Cr had no significant change during treatment. But, serum Cys-C increased and as a result, GFR measured with Cys-C decreased during treatment period. The changes were more considerable in the group of children over 2 years than the group of children under 2 years.

In conclusion, Cys-C is a more reliable method than creatinine for the evaluation of amikacin nephrotoxicity in children with acute pyelonephritis even in a short period of time. Consequently, we recommend the use of Cys-C in any children who was treated with amikacin for evaluation of drug nephrotoxicity.
